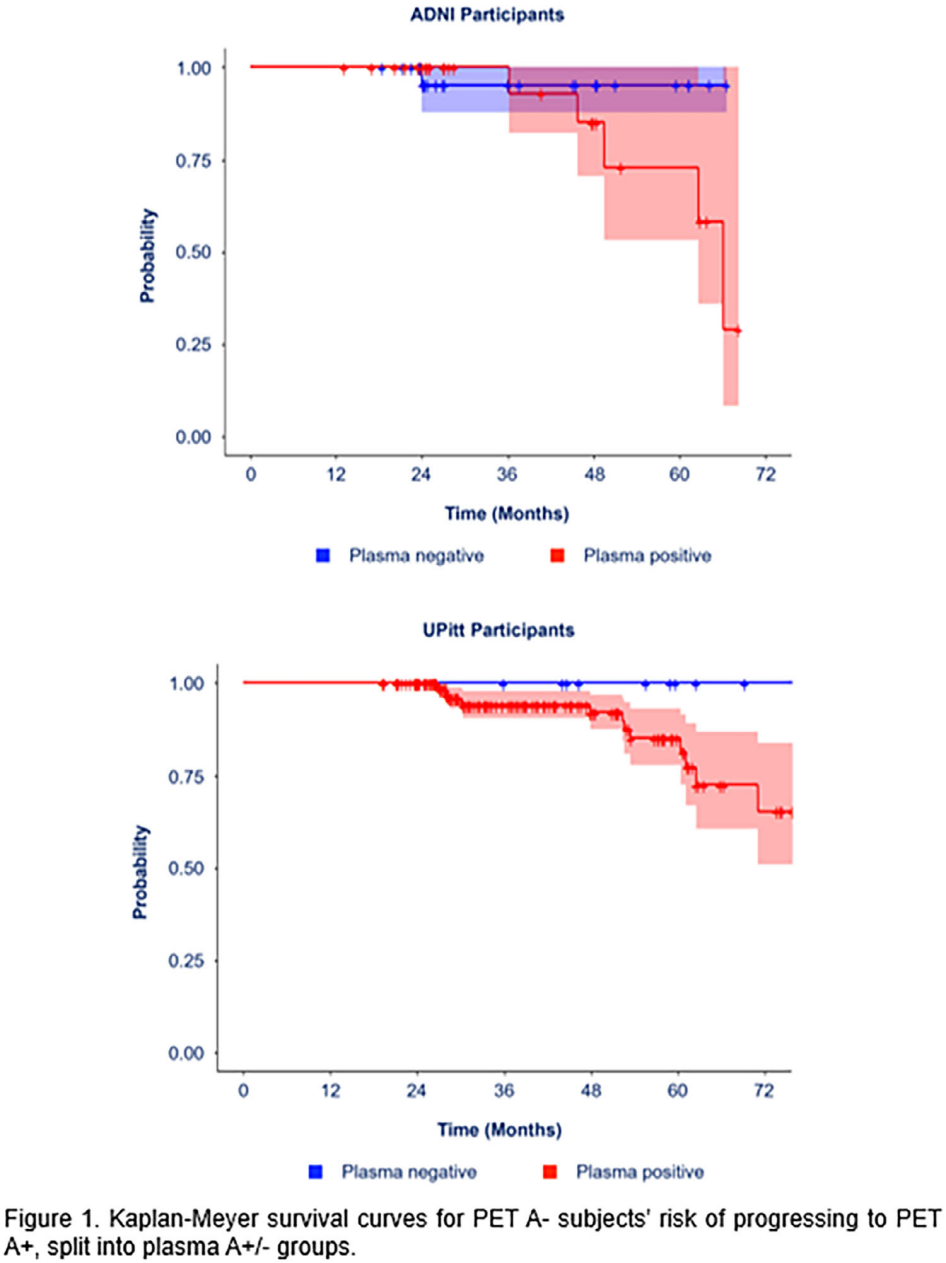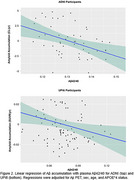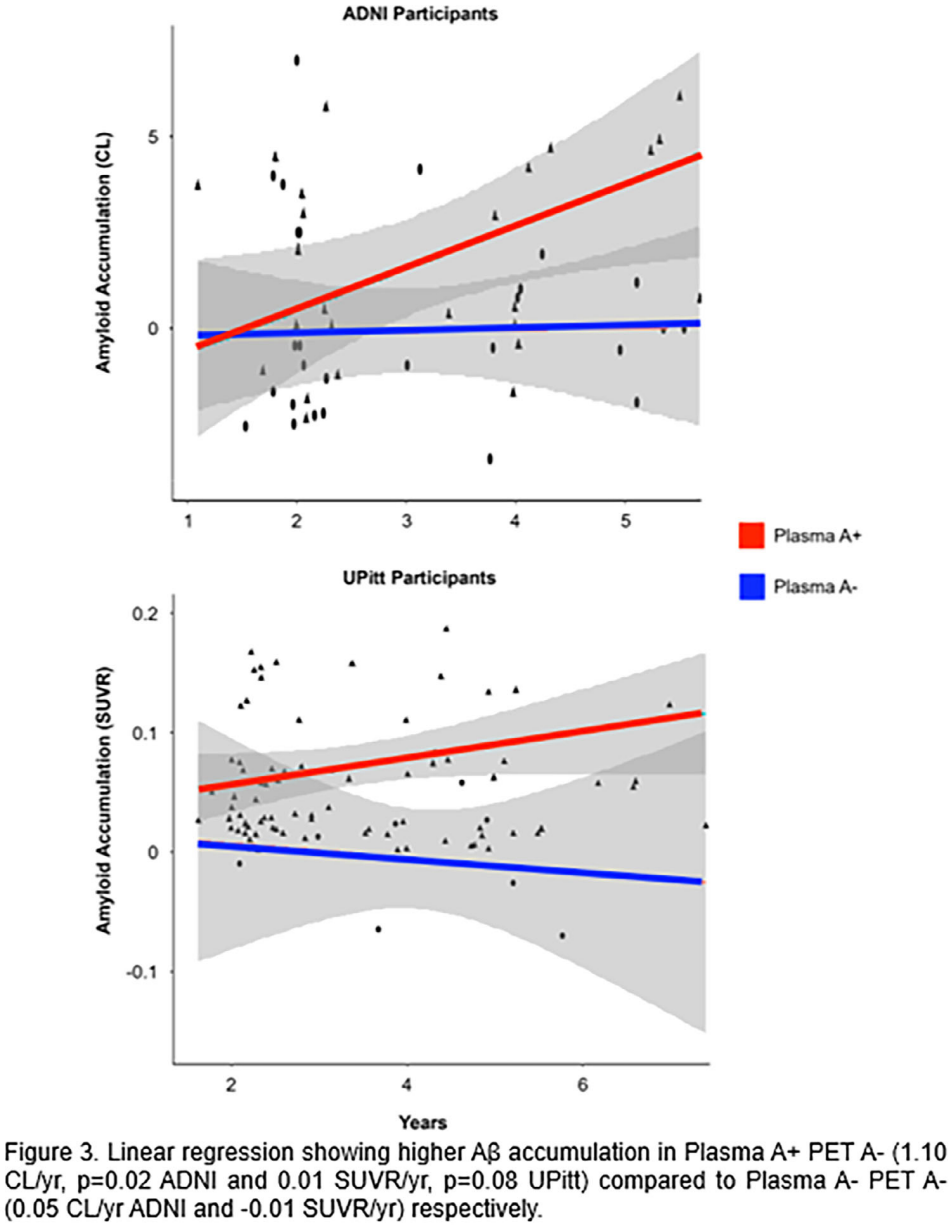# Utilization of plasma Aβ and *p*‐tau217 in predicting Aβ accumulation in PET A‐ non‐demented participants

**DOI:** 10.1002/alz70856_106156

**Published:** 2026-01-11

**Authors:** Alexandra Gogola, Ann D Cohen, Brian J Lopresti, Beth E. Snitz, Milos D. Ikonomovic, Dana L Tudorascu, Davneet S Minhas, Xuemei Zeng, Julia K. Kofler, Cristy Matan, Howard J Aizenstein, Oscar L Lopez, Thomas K Karikari, Victor L. Villemagne

**Affiliations:** ^1^ University of Pittsburgh, School of Medicine, Pittsburgh, PA, USA; ^2^ University of Pittsburgh School of Medicine, Pittsburgh, PA, USA; ^3^ University of Pittsburgh, Pittsburgh, PA, USA; ^4^ The University of Pittsburgh, Pittsburgh, PA, USA

## Abstract

**Background:**

As Alzheimer's disease studies increasingly utilize plasma biomarkers, the associations of plasma and PET biomarkers of β‐amyloid (Aβ) in relation to Aβ accumulation and progression from PET A‐ to A+ must be better understood.

**Methods:**

We evaluated 58 non‐demented participants from ADNI and 170 from the University of Pittsburgh (UPitt) who had plasma Aβ42/Aβ40 measures and longitudinal Aβ‐PET. We additionally evaluated 124 UPitt participants who had *p*‐tau217 measures. All participants were PET A‐ at baseline. Plasma Aβ42/40 values were derived through either the C_2_N mass spectrometry‐based assay (ADNI) or Simoa assay (UPitt). *p*‐tau217 values were derived through the Janssen *p*‐tau217+ Simoa assay. Cut‐off values of 0.15 and 0.11 determined Aβ42/40 status for the ADNI and UPitt datasets, respectively, A cut‐off of 0.083 pg/mL was used for *p*‐tau217. Kaplan‐Meyer survival models evaluated the association of plasma Aβ42/40 status with risk of progression to PET A+. Linear regression models, adjusting for baseline PET Aβ values, sex, age, and APOE*4 status, assessed the association of plasma Aβ42/40 values with Aβ accumulation.

**Results:**

Kaplan‐Meyer survival analysis results showed that plasma Ab status distinctly differentiates PET A‐ trajectories, (Figure 1) with plasma A+ progressing to PET A+ in a median time of 4.0 years in both datasets. Only 3 (2.4%) of the 124 PET A‐ participants presented high *p*‐tau217 values. Linear regression models of the continuous measures revealed that, even with the inclusion Aβ‐PET and the other covariates, plasma Aβ42/40 was significantly associated with Aβ accumulation (ADNI: b=‐142.29, *p* = 0.002; UPitt: b=‐0.27, *p* = 0.02) (Figure 2). Plasma A+ presented significantly higher Aβ accumulation compared to plasma A‐. (Figure 3)

**Conclusions:**

While a better predictor of high Ab deposition than plasma Aβ42/40, *p*‐tau217 is not elevated in PET A‐. On the other hand, plasma Aβ42/40 can predict progression from PET A‐ to A+. This follows the notion that soluble Aβ, measured in plasma or CSF, becomes abnormal before high insoluble Aβ is detectable with PET. Therefore, plasma Aβ‐positive/Aβ‐PET negative identify individuals at the very early stages of Aβ deposition, at risk of progression, requiring a closer follow‐up and likely to benefit the most from anti‐Aβ therapy.